# ﻿﻿﻿﻿﻿﻿﻿﻿﻿﻿﻿﻿﻿﻿﻿Effectiveness of Up-to-Date COVID-19 Vaccination in Preventing SARS-CoV-2 Infection Among Nursing Home Residents — United States, November 20, 2022–January 8, 2023

**DOI:** 10.15585/mmwr.mm7225a4

**Published:** 2023-06-23

**Authors:** Emily Wong, Kira Barbre, Ryan E. Wiegand, Hannah E. Reses, Heather Dubendris, Megan Wallace, Philip Dollard, Jonathan Edwards, Minn Soe, Lu Meng, Andrea Benin, Jeneita M. Bell

**Affiliations:** ^1^Division of Healthcare Quality Promotion, National Center for Emerging and Zoonotic Infectious Diseases, CDC; ^2^Goldbelt C6, Chesapeake, Virginia; ^3^National Center for Immunization and Respiratory Diseases, CDC; ^4^Lantana Consulting Group, East Thetford, Vermont.

Nursing home residents have been disproportionately affected by the COVID-19 pandemic; their age, comorbidities, and exposure to a congregate setting has placed them at high risk for both infection and severe COVID-19–associated outcomes, including death (*1*). Receipt of a primary COVID-19 mRNA vaccination series (*2*) and monovalent booster doses (*3*) have been demonstrated to be effective in reducing COVID-19–related morbidity and mortality in this population. Beginning in October 2022, the National Healthcare Safety Network (NHSN) defined up-to-date vaccination as receipt of a bivalent COVID-19 mRNA vaccine dose or completion of a primary series within the preceding 2 months.* The effectiveness of being up to date with COVID-19 vaccination among nursing home residents in preventing SARS-CoV-2 infection is not known. This analysis used NHSN nursing home COVID-19 data reported during November 20, 2022–January 8, 2023, to describe effectiveness of up-to-date vaccination status (versus not being up to date) against laboratory-confirmed SARS-CoV-2 infection among nursing home residents. Adjusting for calendar week, county-level COVID-19 incidence, county-level social vulnerability index (SVI), and facility-level percentage of staff members who were up to date, up-to-date vaccine effectiveness (VE) against infection was 31.2% (95% CI = 29.1%–33.2%). Nursing home residents should stay up to date with recommended age-appropriate COVID-19 vaccination, which now includes an additional bivalent vaccine dose for moderately or severely immunocompromised adults aged ≥65 years to increase protection against SARS-CoV-2 infection.

The Centers for Medicare & Medicaid Services (CMS) requires CMS-certified nursing homes to submit incident COVID-19 case and vaccination data to NHSN each week.^†^^,^^§^ Data include the number of infections (defined as laboratory-confirmed^¶^ SARS-CoV-2 infections) stratified by patient vaccination status, and the number of residents in the nursing home (with a stay of ≥24 hours) stratified by vaccination status.** During the study period, NHSN defined up-to-date vaccination status as 1) ever having received a COVID-19 mRNA bivalent vaccine dose, or 2) completion of a primary series <2 months earlier. The number of residents who were not up to date was calculated by subtracting the number who were up to date from the total number of residents in the facility. Residents who were not up to date included those who 1) previously received monovalent booster doses but did not receive a bivalent vaccine dose, 2) received the primary series >2 months earlier but did not receive any subsequent doses, 3) received 1 dose of the primary series, or 4) did not receive any COVID-19 vaccine doses.

NHSN analyzed weekly COVID-19 case and up-to-date vaccination status data for CMS-certified nursing homes during November 20, 2022–January 8, 2023. The study period was chosen to coincide with both the inclusion of bivalent vaccine in the definition of up-to-date status and the increase in COVID-19 infections during the winter months.^††^ The study included data submitted by CMS-certified nursing homes that reported both COVID-19 cases and up-to-date vaccination status for each week during the study period.

Analysts merged weekly incident case counts (stratified by up-to-date vaccination status) with weekly resident counts (stratified by up-to-date vaccination status) each week during the study period. Nursing homes that reported no data on up-to-date vaccination status throughout the study period were excluded, as were those that did not meet standard data quality criteria.^§§^ Resident-weeks were calculated by aggregating the number of residents who spent ≥1 day at the facility during the week of data collection over the study period.

The ratio of infection between residents who were up to date and those who were not was determined using a zero-inflated negative binomial mixed model (*4*) to evaluate associations with acquisition of COVID-19, while adjusting for potential confounders. The model used data collected by NHSN and included nursing home as a random effect to account for between-facility variability. Covariates included in models were factors known to be associated with either up-to-date vaccination status or infection, including calendar week, SVI, county-level incidence, and percentage of facility staff members who were up to date with COVID-19 vaccination. VE against infection was estimated as 1 − rate ratio x 100. Analyses were performed using SAS software (version 9.4; SAS Institute) and R software (version 4.0.3; R Foundation). This activity was reviewed by CDC and was conducted consistent with applicable federal law and CDC policy.^¶¶^

The analysis included 108,727 weekly reports from 14,464 nursing homes. Overall, 4,314,714 (48.1%) nursing home resident-weeks were up to date, and 52,853 (40.6%) of COVID-19 patients were up to date. The resulting crude infection rate among up-to-date residents was 12.3 per 1,000 resident-weeks (95% CI = 12.2–12.4) compared with 16.6 per 1,000 resident-weeks (95% CI = 16.5 –16.7) among residents who were not up to date. During the study period, the weekly percentage of residents who were up to date increased from 44.2% to 51.2%.

Each week, COVID-19 incidence among nursing home residents who were up to date (7.6–15.3 cases per 1,000 residents) was lower than incidence among those who were not up to date (11.1–19.1 cases per 1,000 residents) (Figure). The adjusted rate ratio of SARS-CoV-2 infection among residents who were up to date compared with those not up to date was 0.69 (95% CI = 0.67–0.71). Among nursing home residents with up-to-date vaccination, VE against infection was 31.2% (95% CI = 29.1%–33.2%) (Table).

**FIGURE:**
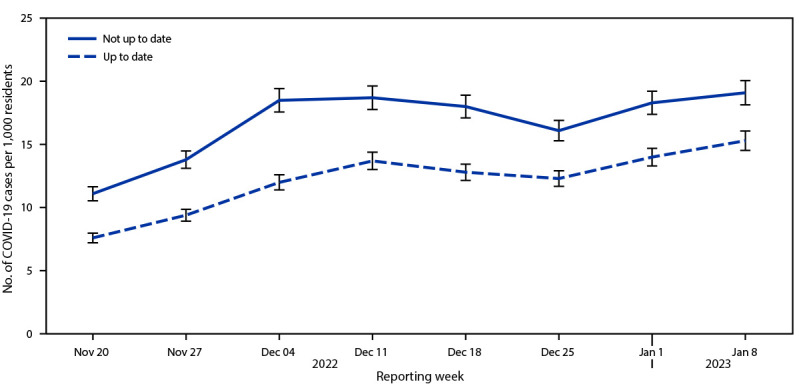
SARS-CoV-2 infections per 1,000 nursing home residents,* by up-to-date vaccination status^†^ and reporting week — National Healthcare Safety Network, United States, November 20, 2022–January 8, 2023 * With 95% CIs indicated by error bars. ^†^ Up-to-date vaccination status was defined as 1) ever having received a bivalent vaccine dose or 2) primary series completed <2 months earlier. The number of residents who were not up to date was calculated by subtracting the number of up-to-date residents from the total number of residents in the facility and included those who 1) received monovalent booster doses but did not receive a bivalent vaccine dose, 2) received the primary series >2 months earlier but did not receive any subsequent doses, 3) received 1 dose of the primary series, or 4) did not receive any COVID-19 vaccine doses.

**TABLE:** Relative effectiveness of being up to date with COVID-19 vaccination in preventing SARS-CoV-2 infection among nursing home residents compared with not being up to date — National Healthcare Safety Network, United States, November 20, 2022–January 8, 2023

Up to date*	No. of resident wks^†^	No. of cases^§^	Crude infection rate (95% CI)^¶^	RR (95% CI)**	VE (%) (95% CI)^††^
No	4,648,119	77,240	16.6 (16.5–16.7)	Ref	Ref
Yes	4,314,714	52,853	12.3 (12.2–12.4)	0.69 (0.67–0.71)	31.2 (29.1–33.2)

**Abbreviations:** Ref = referent group; RR = rate ratio; VE = vaccine effectiveness.

* Up-to-date vaccination status was defined as 1) ever having received a bivalent vaccine dose or 2) completed a primary series <2 months earlier. The number of residents who were not up to date was calculated by subtracting number of up-to-date residents from the total number of residents in the facility and included those who 1) received monovalent booster doses but did not receive a bivalent vaccine dose, 2) received the primary series >2 months earlier but did not receive subsequent doses, 3) received 1 dose of the primary series, or 4) did not receive any COVID-19 vaccine doses.

^†^ Resident-weeks were the number of residents staying in a facility for ≥1 day during the week of data collection aggregated to the study period, stratified by vaccination status reported by nursing homes.

^§^ Cases were the aggregate of weekly case counts stratified by vaccination status reported by nursing homes Cases among residents up to date were defined as infections in residents who became up to date ≥14 days before a positive SARS-CoV-2 test result. Cases who became up to date <14 days before a positive SARS-CoV-2 test result were included in the not up-to-date group.

^¶^ Infections per 1,000 resident-weeks.

** RR results from zero-inflated negative binomial mixed model.

^††^ VE was estimated as 1 − RR x 100.

## Discussion

Among nursing home residents who were up to date with COVID-19 vaccination, VE against SARS-CoV-2 infection was 31.2% during November 20, 2022–January 8, 2023. An analysis of NHSN nursing home data found that during October 10, 2022–January 8, 2023, >99% of residents classified as being up to date had received a bivalent vaccine dose, suggesting that up-to-date vaccination represented the receipt of bivalent vaccine (*5*). During this period, 88% of residents had received ≥1 dose of a primary COVID-19 vaccination series, indicating that most of those who were not up to date had received at least partial vaccination.***

Although this study could not account for waning VE since receiving the bivalent vaccine dose, the VE against infection in this study is similar to other bivalent VE estimates compared with the VE of monovalent vaccine alone against symptomatic infection in adults aged ≥65 years (22%–43%) (*6*), especially considering that this analysis was conducted 2.5–4 months after a bivalent vaccine dose was initially recommended for this population (*7*). In updates on COVID-19 VE presented to CDC’s Advisory Committee on Immunization Practices on February 23, 2023 (*8*) and April 19, 2023 (*9*), bivalent VE against symptomatic infection compared with receipt of monovalent vaccine doses only among immunocompetent adults aged ≥65 years was 38% in the 2 weeks to 1 month after receipt of the bivalent vaccine dose and waned to 21% by 4–5 months after vaccination (*8*). Among symptomatic adults aged ≥65 years who visited an emergency department or urgent care center, bivalent VE against SARS-CoV-2 infection compared with no vaccine was 61% at 7–59 days after the bivalent vaccine dose and waned to 25% at 120–179 days after the bivalent vaccine dose (*9*).

The goal of the U.S. COVID-19 vaccination program is to prevent severe COVID-19–associated outcomes, including death (*7*). Although this study could not assess VE against severe outcomes, VE against severe outcomes for both monovalent (*7*) and bivalent (*10*) mRNA vaccines has been demonstrated to be higher and more sustained than it is against symptomatic infection (*6*). Nonetheless, this analysis of bivalent VE against infection provides important insight into vaccine protection among residents of nursing homes and demonstrates that staying up to date with recommended COVID-19 vaccines protects nursing home residents against SARS-CoV-2 infection.

The findings in this report are subject to at least four limitations. First, the data used in this study include COVID-19 vaccination status and infection but do not include outcomes such as hospitalization and death. Although a meaningful reduction in infection is an important finding, the VE estimate presented in this report does not directly assess the goal of COVID-19 vaccination, which is prevention of severe disease (*7*). Second, because NHSN receives aggregate facility-level data and was not randomized, this analysis could not account for time since vaccination, previous SARS-CoV-2 infection, COVID-19 symptoms, person-level demographic characteristics, or any other potential person-level confounders. Third, the data submitted by facilities to NHSN categorized vaccination status as either up to date or not up to date. The group that was not up to date included persons with a range of vaccination histories. The lack of a comparison group that was naïve to COVID-19 vaccination and infection meant that it was not possible to calculate the VE of up-to-date vaccination compared with no vaccination. The VE calculated by this study represents added benefit of the bivalent vaccine in a largely vaccinated population. Finally, the aggregate data used in this study were reported by nursing homes and could not be verified against patient records. Therefore, misclassification of case and vaccination status of residents is possible.

NHSN provides robust surveillance of vaccination status and SARS-CoV-2 infection among this vulnerable population; these data remain important to assessing the public health impact of changing vaccination guidance. It is important that nursing home residents stay up to date with COVID-19 vaccines and, if eligible, receive an additional bivalent dose to optimize protection against infection and related complications.

**Summary**

**What is already known on this topic?**

Vaccines prevent severe outcomes and staying up to date with recommended COVID-19 vaccination, including receiving a bivalent vaccine dose, provides additional protection against COVID-19 in persons who previously received monovalent vaccines; however, recent data on effectiveness of up-to-date vaccination status among nursing home residents are limited.

**What is added by this report?**

Among nursing home residents who were up to date with COVID-19 vaccination (most had received a bivalent vaccine), vaccine effectiveness against SARS-CoV-2 infection was 31.2%.

**What are the implications for public health practice?**

Staying up to date with COVID-19 vaccination recommendations and, if eligible, receipt of an additional bivalent dose, provides additional protection against SARS-CoV-2 infection. Nursing home residents would benefit from the protection offered by staying up to date with recommended COVID-19 vaccinations.

## Footnotes

* Up-to-date vaccination status was defined as 1) ever having received a bivalent vaccine dose or 2) completed a primary series <2 months earlier. NHSN defines up-to-date vaccination for surveillance purposes at the start of each quarter; the definition has been updated since the study was conducted. https://www.cdc.gov/nhsn/pdfs/hps/covidvax/UpToDateGuidance-508.pdf

†
https://www.cms.gov/files/document/qso-20-29-nh.pdf

§
https://www.federalregister.gov/documents/2021/05/13/2021-10122/medicare-and-medicaid-programs-covid-19-vaccine-requirements-for-long-term-care-ltc-facilities-and

¶
https://www.cdc.gov/nhsn/pdfs/covid19/ltcf/57.144-toi-508.pdf

**
https://www.cdc.gov/nhsn/forms/instr/COVIDVax.LTC_.Residents.TOI_.MAY2022-508.pdf

††
https://covid.cdc.gov/covid-data-tracker/#trends_weeklycases_select_00

§§ Data were excluded based on the following conditions: 1) the number of residents was equal to or more than the number of beds occupied or <75% of beds were occupied (295 facilities excluded); 2) facility coverage for ≥1 dose was <60% (518 facilities excluded), because low partial vaccination coverage indicates incorrect reporting or an atypical facility; 3) the number of up-to-date residents was >10 in the previous week, and the number of up-to-date residents declined by >50% in the current week (1,905 facilities excluded), because a large percent decline indicates incident, rather than cumulative, reporting in large facilities.

¶¶ 45 C.F.R. part 46, 21 C.F.R. part 56; 42 U.S.C. Sect. 241(d); 5 U.S.C. Sect. 552a; 44 U.S.C. Sect. 3501 et seq.

***
https://covid.cdc.gov/covid-data-tracker/#vaccinations-nursing-homes
